# The Prognostic Utility of Lymphocyte-Based Measures and Ratios in Chemotherapy-Induced Febrile Neutropenia Patients following Granulocyte Colony-Stimulating Factor Therapy

**DOI:** 10.3390/medicina58111508

**Published:** 2022-10-24

**Authors:** Omar M. Halalsheh, Yazan O. Al Zu’bi, Ahmed H. Al Sharie, Farouk H. Wafai, Nadeem Alabdallah, Jumana AlSeidi, Alia A. Hussein, Majd N. Daoud, Abubaker A. Malkawi, Ahmad O. Alomari, Osama Alshari

**Affiliations:** 1 Division of Urology, Department of General Surgery and Urology, Faculty of Medicine, Jordan University of Science & Technology, Irbid 22110, Jordan; 2 Faculty of Medicine, Jordan University of Science & Technology, Irbid 22110, Jordan; 3 Division of Oncology, Department of Internal Medicine, Faculty of Medicine, Jordan University of Science & Technology, Irbid 22110, Jordan

**Keywords:** febrile neutropenia, chemotherapy, lymphopenia, lymphocyte ratios, granulocyte colony-stimulating factor, G-CSF

## Abstract

*Background and Objectives:* Chemotherapy-induced febrile neutropenia is the most widespread oncologic emergency with high morbidity and mortality rates. Herein we present a retrospective risk factor identification study to evaluate the prognostic role of lymphocyte-based measures and ratios in a cohort of chemotherapy-induced febrile neutropenia patients following granulocyte colony-stimulating factor (G-CSF) therapy. *Materials and Methods*: The electronic medical records at our center were utilized to identify patients with a first attack of chemotherapy-induced febrile neutropenia and were treated accordingly with G-CSF between January 2010 to December 2020. Patients’ demographics and disease characteristics along with laboratory tests data were extracted. Prognosis-related indicators were the absolute neutrophil count (ANC) at admission and the following 6 days besides the length of stay and mortality rate. *Results*: A total of 80 patients were enrolled, which were divided according to the absolute lymphocyte count at admission into two groups, the first includes lymphopenia patients (*n* = 55) and the other is the non-lymphopenia group (*n* = 25) with a cutoff point of 700 lymphocytes/μL. Demographics and baseline characteristics were generally insignificant among the two groups but the white blood cell count was higher in the non-lymphopenia group. ANC, neutrophils percentage and ANC difference in reference to admission among the two study groups were totally insignificant. The same insignificant pattern was observed in the length of stay and the mortality rate. Univariate analysis utilizing the ANC difference compared to the admission day as the dependent variable, revealed no predictability role in the first three days of follow up for any of the variables included. However, during the fourth day of follow up, both WBC (OR = 0.261; 95% CI: 0.075, 0.908; *p* = 0.035) and lymphocyte percentage (OR = 1.074; 95% CI: 1.012, 1.141; *p* = 0.019) were marginally significant, in which increasing WBC was associated with a reduction in the likelihood of ANC count increase, compared to the lymphocyte percentage which exhibited an increase in the likelihood. In comparison, sequential ANC difference models demonstrated lymphocyte percentage (OR = 0.961; 95% CI: 0.932, 0.991; *p* = 0.011) and monocyte-to-lymphocyte ratio (OR = 7.436; 95% CI: 1.024, 54.020; *p* = 0.047) reduction and increment in the enhancement of ANC levels, respectively. The fifth day had WBC (OR = 0.790; 95% CI: 0.675, 0.925; *p* = 0.003) to be significantly decreasing the likelihood of ANC increment. *Conclusions:* we were unable to determine any concrete prognostic role of lymphocyte-related measures and ratios. It is plausible that several limitations could have influenced the results obtained, but as far as our analysis is concerned ALC role as a predictive factor for ANC changes remains questionable.

## 1. Introduction

Amidst the daunting side effects of receiving chemotherapy for solid tumors, febrile neutropenia (FN) remains the most widespread and lethal complication arising in 10–20% of patients undergoing treatment [1]. FN delays the continuation of chemotherapy until the neutropenia subsides, increasing treatment cost, prolonging hospitalization, and reducing the efficacy of chemotherapy as it allows chemo-resistant clones to emerge and cancer cells to recover [2]. With the continued increase in the number of chemotherapy use, FN poses as a major obstacle in the development efforts of potential cures to lethal malignancies [1]. The American Society of Clinical Oncology (ASCO) and the Infectious Disease Society of America (IDSA) established joint guidelines that diagnose neutropenia when absolute neutrophil counts (ANC) are less than 1000/μL), and diagnose FN when the mentioned ANC count is associated with an oral temperature ≥ 38.0 °C persisting over a span of one hour [3]. This specific definition of FN has been accepted and adopted by the American Society of Clinical Oncology (ASCO), American Society of Hematology (ASH), and National Comprehensive Cancer Network (NCCN) [3]. However, there is some flexibility globally as the European Society of Medical Oncology (ESMO) characterizes neutropenia with ANC counts under 500/μL) [4].

During the course of malignancy, neutropenia occurs most prominently in the early periods of active treatment due to severe therapy-induced myelosuppression but could still occur at earlier stages from initial diagnosis or later at end-stage disease [3]. Additionally, the fever associated with FN has been hypothesized to be the result of a complex reaction of the innate immune system between molecules expressed by micro-bacterial pathogens, known as pathogen-associated molecular patterns (PAMPS) and damage-associated molecular patterns (DAMPS), which are derived from within tissue as a consequence of mucosal barrier injury [5]. Even though fever could be the sole marker of infection in these high-risk patients, sterile inflammation has been found in 40% of this group [3]. Furthermore, inflammatory signs and symptoms are thought to be reduced in febrile neutropenic patients due to the deficiency of inflammatory cytokines such as IL-1β, IL-12, TNF-α, and IL-6 and anti-inflammatory cytokines such as IL-10; all together preventing the physiological feedback inflammatory process, intensifying the damage [5].

The introduction of antibacterial, antifungal and antiviral agents favorably influences the prognosis, morbidity and mortality in febrile neutropenic patients [6]. When these antimicrobial therapies are administered at the onset of fever, it radically transforms the successful management of the infection; in fact, a delay in treatment of Gram-negative infections in particular has shown to increase mortality rates to up to 70% compared to 4%-20% in patients who have been treated early on [7,8]. However, due to the inadequate immune response with FN, it is hard to identify the presence of the infection with symptoms excluding fever being absent [7]. The high mortality rates associated with first waiting to identify the infecting organisms before the application of antimicrobial agents led to the abandoning of this strategy and the exploration of leading causes of infection [7]. Up until the 1980s, Gram-negative bacilli such as *Escherichia coli, Klebsiella species, Enterobacter and Pseudomonas aeruginosa* were the leading cause of bacterial infections [8,9]. Since then, there has been a transition in bacilli dominance, hypothesized to be a cause of an increased utilization of indwelling catheters, that caused the Gram-positive bacilli such as *coagulase-negative staphylococcus, Staphylococcus aureus (including methicillin-resistant Staphylococcus aureus), Enterococcus, Streptococcus viridans, Streptococcus pneumoniae, and Streptococcus pyogenes* to be the dominant infecting organisms [9]. According to this finding, the cornerstone of antimicrobial agents to be administered at the onset of infection was decided to be antibiotics. This includes broad-spectrum cephalosporin with antipseudomonal activity, carbapenem, or extended-spectrum penicillin. Patients who have severe penicillin allergy or those who experience complications are given ciprofloxacin plus clindamycin or aztreonam plus vancomycin. Even though the prophylactic use of fluoroquinolones has shown to reduce rates of infections due to Gram-negative rods, many studies did not demonstrate a decrease in overall mortality but on the other hand observed the development of multi-drug resistant organisms; hence, prophylaxis strategies vary at different treatment centers [9,10].

The administration of the cytokine granulocyte colony-stimulating factor (G-CSF) has proven to alleviate chemotherapy-induced neutropenia, neutropenia associated with hematopoietic stem cell transplantation and severe chronic neutropenia [11]. G-CSF treatment drastically cuts down on neutropenia’s duration in patients that develop it due to post solid organ transplant complications and it attenuates their risk of allograft loss and death without causing any effects on acute cellular rejection [12]. The food and drug administration (FDA) has already approved the wide use of G-CSF and granulocyte/macrophage-colony-stimulating factor (GM-CSF), benefiting 20 million people worldwide and saving USD 5 billion per year annually in the USA, for the treatment of congenital and acquired neutropenias where GM-CSF is typically recommended for neutropenia associated with stem cell transplantation as it also promotes antigen presenting cells function [13]. In the current study, we evaluate the prognostic role of lymphopenia-related measures and ratios in chemotherapy-induced FN patients treated with G-CSF.

## 2. Materials and Methods

### 2.1. Study Design

This study represents a comprehensive and inclusive analysis of a previously reported cohort of patients [12]. In this extended analysis we aimed to study the prognostic effects of absolute lymphocyte count (ALC) in chemotherapy-induced febrile neutropenia patients subsequently receiving G-CSF in the acute setting. Lymphocyte percentage, monocyte-to-lymphocyte ratio (MLR), and platelet-to-lymphocyte ratio (PLR) have been studied as well. The data collection process has been precisely described along with a detailed inclusion and exclusion criteria previously [12]. In addition to the patient’s demographic data, and their baseline disease characteristics, the complete blood count (CBC) and white blood cell (WBC) differential were extracted at admission and the subsequent 6-day follow-up period post admission. Further collection was unreasonable due to the lack of significant data for valuable statistical analysis. The total number of enrolled patients was 80, which have been divided according to the lymphocyte count at admission into two groups: the first includes lymphopenia patients (*n* = 55), with a lymphocyte count ≤ 700 cells μL, while the other is the non-lymphopenia group (*n* = 25) with an absolute count of > 700 cells μL. Outcome variables included the hospitalization period in days, mortality, the ANC difference at each follow up period compared to admission and a sequential ANC difference; in which ANC levels at each follow up day compared to the previous one was calculated. Subsequently, ANC difference was transformed into binary data where a decrease or no change was designated, the value 0 being defined as “Did not improve”, while an increase in ANC was designated a value of 1, defined as “improved”. This study was performed in accordance with the Declaration of Helsinki, taking into consideration all the ethical regulations of King Abdullah University Hospital (KAUH). Patients’ data was analyzed in aggregates ensuring anonymity, hence the need for consent from patients was alleviated.

### 2.2. Statistical Analysis

Data was analyzed using IBM SPSS statistical package for Windows v.26 (Armonk, NY, USA). A *p* ≤ 0.05 was considered statistically significant. Normally distributed continuous variables were represented as mean ± standard deviation of the mean, while non-normally distributed data was presented as median (interquartile range [IQR]). Categorical data was presented as frequency (percentage). Normality was tested using the Shapiro–Wilk test. Chi-square test, Fisher’s exact test or likelihood ratio were used to compare categorical variables accordingly. Non-parametric variables among the study groups were compared using Mann–Whitney *U*-test. On the contrary, independent *t*-test was used for parametric ones. Association of CBC variables and the ANC difference (compared to admission and sequential difference) is determined using univariate binary logistic analysis. Consequently, any significant variable was used in the multivariable regression model. Day 6 was eliminated from the univariate and multivariable analysis due to the low number of patient records that could be retrieved.

## 3. Results

The analysis incorporated 80 patients, categorized into two groups in accordance with the absolute lymphocyte count: a lymphopenia (*n* = 55) versus a non-lymphopenia (*n* = 25) group. The lymphopenia group was generally younger with a male-to-female ratio of 1:1. Table 1 lists the demographics and baseline characteristics, which were generally insignificant among the two groups. Table 2 illustrates the CBC parameters and indices along the six days of follow up. Despite the insignificance the WBC was higher in the non-lymphopenia group. Unfortunately, ANC, neutrophil percentage and ANC difference in reference to admission among the two study groups were totally insignificant during the follow up period as illustrated in Table 3. The length of stay and the mortality rate were not statistically different among the test groups. Notwithstanding the insignificance, generally the non-lymphopenia group had greater ANC levels and ANC increment among the follow-up period. Univariate analysis utilizing the ANC difference compared to the admission day as the dependent variable (Table 4), revealed no predictability role in the first three days of follow up for any of the variables included. However, during the fourth day of follow up, both WBC (OR = 0.261; 95% CI: 0.075, 0.908; *p* = 0.035) and lymphocyte percentage (OR = 1.074; 95% CI: 1.012, 1.141; *p* = 0.019) were marginally significant, in which increasing WBC was associated with a reduction in the likelihood of ANC count increase, compared to the lymphocyte percentage, which exhibited an increase in the likelihood. In comparison, sequential ANC difference models (Table 5) demonstrated lymphocyte percentage (OR = 0.961; 95% CI: 0.932, 0.991; *p* = 0.011) and MLR (OR = 7.436; 95% CI: 1.024, 54.020; *p* = 0.047) reduction and increment in the enhancement of ANC levels, respectively. Fifth day had WBC (OR = 0.790; 95% CI: 0.675, 0.925; *p* = 0.003) to be significantly decreasing the likelihood of ANC increment. It is evident that sequential ANC models, compared to ANC difference in reference to the admission day models, yielded different results. Finally, we were unable to determine any concrete prognostic role of ALC or any of the indices included in the study. It is plausible that several limitations could have influenced the results obtained, but as far as our analysis is concerned, ALC role as a predictive factor for ANC changes remains questionable.

## 4. Discussion

Risk assessment is utilized today in virtually all medical settings to help guide our approach to patient care and ultimately lead to the most favorable outcome. The importance of risk factor identification has been demonstrated multifariously in medicine, and is often single-handedly responsible for the choice of diagnostic work-ups, screening efforts, and acute management. Risk assessment, in its most basic form in medicine, revolves around understanding the correlation between independent risk factors and how they relate to different disease processes. Robust clinically relevant correlations are found aplenty in medicine. Grouping multiple risk factors and markers together, and giving each of them a numerical value, gave rise to scoring systems that can be used to predict risk of developing or acutely presenting with specific pathologies, and their respective morbidities and mortalities. These scoring systems not only provide a more objective measure for the diagnosis, but also guide the laboratory testing, imaging, and management. Risk factor identification and risk assessment is as important and just as applicable in oncologic diagnostics, prevention, and management.

FN is one the most common oncologic emergencies today. FN is defined as an ANC < 0.5 × 10^9^/L (500 cells/mm^3^) accompanied by a fever of > 38.3 °C or >38.0 °C sustained for 60 min [13]. FN occurs in the setting of cancer patients treated by myelosuppressive chemotherapy, whereby the effect of the chemotherapy leaves the patient in a neutropenic state and at an elevated risk of infections. The length and number of chemotherapeutic cycles, length of neutropenic state, and presence of comorbidities have all been shown to increase the risk of developing FN [14]. Overall incidence of FN in US cancer patients was found to be 91,560 cases (5.9% incidence) in 2012 [15], in the UK it is reported to be 1.94% of all oncologic admissions [16], and 2% among solid organ tumors in Spain [17]. The timely diagnosis and treatment of FN is of utmost importance due to the extremely high mortality rate, length of stay (LOS), and additionally cost associated with delayed treatment in these patients. The overall mortality associated with FN ranges from 2.6–50.6% [18], with the mortality rate for untreated FN cases being reported as high as 90% [19]. The increased mortality is associated with increased LOS and overall cost of treatment [20], these being the natural sequelae of patients who had poorer outcomes overall requiring longer hospitalization and vigorous therapies. Furthermore, from a healthcare cost perspective, the outpatient management of FN, which is reserved for low-risk FN patients, is dramatically more cost-effective than inpatient management. In a study by Elting et al., the cost of therapy for FN in the outpatient setting was found to be approximately half as much as it was in the inpatient setting ($7.7k vs. $15k) [21]. Since FN was brought upon as a complication of the chemotherapeutic treatment the patient received for their cancer, this entails that the chemotherapeutic regimen must be suspended till the patient clears the infection. Hence, the incidence of FN is associated with a delay in treatment for cancer patients, possibly accruing, indirectly, an increased risk of mortality and morbidity from the primary malignancy. For all the aforementioned mentioned reasons, correctly diagnosing and treating FN as early on as possible is critical and carries significant implications for the patients’ mortality, morbidity, and healthcare costs.

To this purpose, multiple scoring systems have been established and externally validated. Talcott et al. proposed the first risk-stratification system in 1988 [22], providing an objective measure for risk assessment of FN outcomes and severity using relevant risk factors. Just over a decade later, the Multinational Association of Supportive Care of Cancer (MASCC) score was developed [23]. The authors studied the correlation between multiple patient characteristics and the resolution of FN without complications and found the sensitivity of the MASCC score to be significantly higher than Talcott’s system (71% vs. 30%), but with MASCC score having a lower specificity than Talcott’s system (68% v 90%). However, further research validating the MASCC score has shown it to have a higher sensitivity (95%) and specificity (95%) than in the original paper [24], showing that it is probably a more accurate method of classifying FN patients into high-risk and low-risk groups, hence, it became the most widely used method for assessing FN patients today. In 2015, however, a new risk-stratification system, called the Clinical Index of Stable Febrile Neutropenia (CISNE) score [25], was proposed to be more sensitive and specific than its predecessors.

The utility of lymphocyte counts and ratios as prognostic factors for FN in oncologic patients receiving chemotherapy has been demonstrated in a number of studies in the medical literature. The ALC, PLR, and NLR have all been reported as useful prognostic factors that are derived from lymphocyte values to predict severity, mortality rates, LOS, and length of antibiotic management in FN. In 1996, Blay et al. demonstrated that a 5-day lymphocyte count of less than 700/µL to be an independent risk factor for FN [26]. They found that 49% of patients with a 5-day lymphocyte count of less than 700/µL in the study developed FN, while only 10% of patients with an ALC higher than 700/µL developed FN. These results have been validated by subsequent peer-reviewed studies demonstrating that both pretreatment lymphopenia, as well as during and post-treatment values, were good predictors of the risk of FN [27,28,29]. In 2008, Jenkins et al. proposed a classification system for neutropenic events (NE) [27] consisting of five groups based on the pretreatment ANC and ALC of the patients. Patients in Group 1 of the Jenkins study (ANC > 5.2 × 10^9^/L and ALC > 2.4 × 10^9^/L), had an incidence of 18% and 4% of NE and FN, respectively, while Group 5 (ANC < 3.1 × 10^9^/L and ALC < 1.5 × 10^9^/L) had an incidence of 52% and 21% of NE and FN, respectively. However, Chen et al. (2014) have challenged the Jenkins system [30], showing in their paper that the Jenkins system is not capable of identifying high risk patients with sufficient accuracy. Interestingly, they found that the addition of the pretreatment AMC as a predictor to the Jenkins model allowed them to identify a subgroup of patients with a significantly high risk of FN (23.1%). The totality of the studies has shown a higher sensitivity of 5-day ALC than 1st day ALC as a predictor for FN. This may indicate that while the ALC is a useful predictor of FN, it may not be best used independently as an early predictor of FN at the start of chemotherapy, but may be best used in conjunction with other predictive factors such as AMC. Aside from ALCs, the use of lymphocyte ratios is another promising and potentially valuable tool as a prognostic factor in FN, albeit literature is sparse. These ratios allow for the inclusion of additional parameters along with the ALC which may provide better prognostic outcomes in clinical use. The NLR has been shown to be a simple index of systemic inflammatory response in inflammatory diseases and some malignancies [31]. Chantharakhit et al. conducted a study to assess the utility of pretreatment NLR as a prognostic factor for FN in a cohort of Thai females with breast cancer. After adjusting for confounding factors and establishing a cut-off point for the NLR, the researchers found that a pretreatment NLR > 2.4 had a sensitivity and specificity of 66.67% and 64.47%, respectively, with the risk of FN for patients with the cut-off pretreatment NLR of > 2.4 being 2.810 times, which was found to be statistically significant (*p* = 0.038). Another lymphocyte-based ratio that has some evidence in the literature supporting its use as a prognostic factor for mortality in FN is the PLR.

In a study by Kim et al. [32], the use of C-reactive protein (CRP), immature granulocyte count, white blood cell (WBC) count, ANC, NLR, and PLR were used as prognostic factors for 1-month mortality in septic shock patients with FN at admission and after G-CSF administration. At admission, the observed inflammatory markers were not found to have significant prognostic value. After G-CSF administration, however, the PLR was found to be an independent prognostic factor for 1-month mortality in the cohort. The optimal cut-off value for survival was found to be a PLR > 100. A PLR > 100 after the administration of G-CSF was predictive of 1-month survival with a sensitivity and specificity of 89.4% and 46.2%, respectively. Although the use of lymphocyte-based prognostic factors appears to be useful in identifying risk of FN and risk of mortality in FN, these ratios are not without issue.

Regarding the successfulness of G-CSF therapy, the single most important independent predictive factor is the recovery of a neutrophil count of at least 0.5 × 10^9^/L [33]. Other factors that have been shown to predict the successfulness of G-CSF prophylaxis include the prophylactic intensity of G-CSF therapy, history of chemotherapy-induced neutropenia (CIN), and female gender. Aapro et al. looked at predictive factors for CIN and FN in patients undergoing prophylactic G-CSF therapy, and assessed their predictive utility at the patient-level (assessment of risk factors, determinants, and predictors before the first cycle of chemotherapy) and cycle-level (reassessment of risk at the beginning of each cycle) [34]. The study found that the prophylactic intensity, being divided into under-, correctly, or over-prophylacted relative to current European Organization for Research and Treatment of Cancer (EORTC) guidelines, was a major determinant for G-CSF therapy success. Under-prophylaxis was found to be a predictor of negative outcomes, while over-prophylaxis, interestingly, was found to negate the probability of negative outcomes. The predictive ability of prophylactic intensity was similar and found to be significant in both the patient-level and cycle-level analysis, further corroborating its significance. Furthermore, concomitant antibiotic prophylaxis was found to correlate with an increased risk of negative events, while old age and history of anemia were not found to affect the risk. These results of concomitant antibiotic prophylaxis, age, and history of anemia contradict the established literature regarding their relation to FN, and are likely to be the result of physician vigilance.

Finally, the current study has quite a few limitations that might have influenced the results observed, including the small sample size and the retrospective nature of the study, along with the acquisition of data from patient charts over a long stretch of time resulting in dealing with inconsistent charting as a result of human error and changes in in-house protocols over time.

## 5. Conclusions

In conclusion, we were unable to determine any concrete prognostic role of lymphocyte-related measures and ratios. It is plausible that several limitations could have influenced the results obtained, but as far as our analysis is concerned, ALC role as a predictive factor for ANC changes remains questionable.

## Figures and Tables

**Table 1:** Demographics and disease characteristics of a cohort of patients with chemotherapy-induced febrile neutropenia treated with G-CSF and subdivided according to the absolute lymphocyte count at admission with a cutoff point of 700 lymphocytes/μL^−1^.

Variable	Lymphopenia (*n* = 55)	Non-Lymphopenia (*n* = 25)	*p*-Value
**Age (Year)**			0.068
Median (IQR)	34.0 (41.0)	45.0 (35.0)	
**Gender**			0.952
Male	26.0 (47.3)	12.0 (48.0)	
Female	29.0 (52.7)	13.0 (52.0)	
**Malignancy**			0.275
Hematological	27.0 (49.1)	9.00 (36.0)	
Solid	28.0 (50.9)	16.0 (64.0)	
**Distant metastasis**			0.113
Yes	9.00 (16.4)	8.00 (32.0)	
No	46.0 (83.6)	17.0 (68.0)	
**Chemotherapy cycles**			0.754
Median (IQR)	3.00 (3.00)	2.00 (3.00)	
**Bone marrow involvement**			0.744
Yes	9.00 (16.4)	3.00 (12.0)	
No	46.0 (83.6)	22.0 (88.0)	
**Chemotherapy regimens**			0.133
ABVD	3.00 (5.50)	2.00 (8.00)	
AC	5.00 (9.10)	-	
Hyper-CVAD	3.00 (5.50)	-	
ICE	3.00 (5.50)	1.00 (4.00)	
Paclitaxel	2.00 (3.60)	1.00 (4.00)	
R-CHOP	8.00 (14.5)	4.00 (16.0)	
R-ICE	2.00 (3.60)	1.00 (4.00)	
VCD	6.00 (10.9)	-	
Others	23.0 (41.8)	16.0 (64.0)	

IQR, interquartile range; ABVD, adriamycin, bleomycin, vinblastine, and dacarbazine; AC, adriamycin, and cyclophosphamide; Hyper-CVAD, hyperfractionated cyclophosphamide, vincristine, doxorubicin, and dexamethasone; ICE, ifosfamide, carboplatin, and etoposide; R-CHOP, rituximab, cyclophosphamide, daunorubicin, vincristine, and prednisone; R-ICE, rituximab, ifosfamide, carboplatin, and etoposide; VCD, bortezomib, cyclophosphamide, and dexamethasone.

**Table 2:** The difference in WBC count, MLR, and PLR among test groups at different follow up points.

Variable	Lymphopenia (*n* = 55)	Non-Lymphopenia (*n* = 25)	*p*-Value
**Admission**			
WBC	0.676 ± 0.437	1.85 ± 0.652	0.000 *
MLR	0.333 (0.696)	0.274 (0.342)	0.269
PLR	0.382 (0.427)	0.185 (0.117)	0.000 *
**Day 1**			
WBC	1.44 ± 1.97	2.46 ±1.55	0.038 *
MLR	0.541 (1.29)	0.303 (0.369)	0.118
PLR	0.263 (0.377)	0.150 (0.121)	0.001 *
**Day 2**			
WBC	3.95 ± 4.93	6.87 ± 6.86	0.037 *
MLR	0.758 (1.52)	0.443 (0.524)	0.048 *
PLR	0.178 (0.294)	0.127 (0.0779)	0.005 *
**Day 3**			
WBC	4.85 (10.5)	9.70 (20.3)	0.058
MLR	0.594 (1.40)	0.450 (0.553)	0.187
PLR	0.172 (0.254)	0.104 (108)	0.002 *
**Day 4**			
WBC	10.1 ± 11.4	9.36 ± 8.02	0.828
MLR	0.889 (1.41)	0.384 (0.512)	0.006 *
PLR	0.106 (0.165)	0.0953 (0.0471)	0.474
**Day 5**			
WBC	4.02 (8.32)	12.8 (12.5)	0.184
MLR	1.42 (1.71)	0.500 (0.428)	0.034 *
PLR	0.145 (0.181)	0.0778 (0.0648)	0.071
**Day 6**			
WBC	8.26 ± 6.90	10.8 ± 8.21	0.479
MLR	0.833 (0.758)	0.683 (0.436)	0.782
PLR	0.0804 (0.136)	0.153 (0.0989)	0.405

WBC, white blood cell count; MLR, monocyte-to-lymphocyte ratio; PLR, platelet-to-lymphocyte ratio. * Denotes statistical significance.

**Table 3:** Absolute neutrophil count, neutrophil percentage and absolute neutrophil count difference (admission as a reference) among test groups at different follow up points.

Variable	Lymphopenia (*n* = 55)	Non-Lymphopenia (*n* = 25)	*p*-Value
**Admission**			
ANC	147 ± 185	19.5 ± 16.5	0.002 *
Neutrophils (%)	17.6 ± 14.6	425 ± 395	0.596
**Day 1**			
ANC	693 ± 1581	807 ± 1121	0.764
Neutrophils (%)	22.3 ± 22.2	24.9 ± 17.7	0.629
ANC difference	53.0 (138)	170 (518)	0.728
**Day 2**			
ANC	2459 ± 4233	3866 ± 4342	0.183
Neutrophils (%)	39.5 ± 26.8	41.7 ± 24.7	0.738
ANC difference	482 (3055)	2627 (4740)	0.294
**Day 3**			
ANC	2747 (7968)	6401 (12,217)	0.213
Neutrophils (%)	51.7 ± 29.7	45.3 ± 26.7	0.413
ANC difference	2625 (7913)	6155 (11,420)	0.496
**Day 4**			
ANC	7294 ± 8589	6586 ± 7141	0.788
Neutrophils (%)	57.5 ± 27.9	49.5 ± 32.0	0.396
ANC difference	3858 (12,511)	3345 (12,243)	0.650
**Day 5**			
ANC	2880 (7067)	5100 (10,587)	0.648
Neutrophils (%)	56.9 ± 28.2	47.8 ± 28.3	0.396
ANC difference	2873 (7096)	4650 (11,218)	0.921
**Day 6**			
ANC	5791 ± 5337	8005 ± 7482	0.541
Neutrophils (%)	56.8 ± 32.1	64.1 ± 17.6	0.590
ANC difference	4324 (9418)	2451 (14,525)	0.501
**Hospitalization period**	5.00 (3.00)	5.00 (2.50)	0.591
**Mortality rate**	25.00%	34.15%	0.595

ANC, absolute neutrophil count. * Denotes statistical significance.

**Table 4:** Univariate and multivariate analysis of different measures and ratios in predicting the absolute neutrophil count difference (admission as a reference).

Variable	OR (95% CI)			
	Univariate Analysis	*p*-Value	Multivariable Analysis	*p*-Value
**Day 1**				
WBC	1.07 (0.549–2.07)	0.850	NA	NA
ALC	1.00 (0.999–1.00)	0.937	NA	NA
Lymphocyte percentage	0.997 (0.972–1.02)	0.846	NA	NA
MLR	3.29 (0.754–14.3)	0.113	NA	NA
PLR	0.978 (0.142–6.74)	0.982	NA	NA
**Day 2**				
WBC	0.933 (0.441–1.98)	0.857	NA	NA
ALC	1.00 (0.999–1.00)	0.992	NA	NA
Lymphocyte percentage	1.00 (0.976–1.03)	0.812	NA	NA
MLR	1.17 (0.465–2.96)	0.736	NA	NA
PLR	1.88 (0.193–18.3)	0.588	NA	NA
**Day 3**				
WBC	0.732 (0.291–1.84)	0.732	NA	NA
ALC	1.00 (0.998–1.00)	0.794	NA	NA
Lymphocyte percentage	1.02 (0.981–1.06)	0.343	NA	NA
MLR	228 (0.728–71,413)	0.064	-	-
PLR	3.34 (0.082–136)	0.524	NA	NA
**Day 4**				
WBC	0.261 (0.075–0.908)	0.035 *	0.302 (0.075–1.22)	.093
ALC	0.999 (0.997–1.00)	0.503	NA	NA
Lymphocyte percentage	1.07 (1.01–1.14)	0.019	1.08 (1.01–1.16)	.034
MLR	29.6 (0.040–21,877)	0.315	NA	NA
PLR	9.85 (0.079–1224)	0.353	NA	NA
**Day 5**				
WBC	0.306 (0.079–1.18)	0.085	NA	NA
ALC	0.999 (0.997–1.00)	0.429	NA	NA
Lymphocyte percentage	1.07 (1.00–1.14)	0.045 *	-	-
MLR	5.55 (0.018–1691)	0.557	NA	NA
PLR	75.8 (0.033–172,954)	0.273	NA	NA

WBC, White blood cell count; ALC, absolute lymphocyte count; MLR, monocyte-to-lymphocyte ratio; PLR, platelet-to-lymphocyte ratio; NA, not applicable. * Denotes statistical significance.

**Table 5:** Univariate and multivariate analysis of different measures and ratios in predicting the absolute neutrophil count difference (sequential).

	OR (95% CI)			
Variable	Univariate Analysis	*p*-Value	Multivariable Analysis	*p*-Value
**Day 2**				
WBC	1.24 (0.817–1.90)	0.309	NA	NA
ALC	1.00 (0.999–1.00)	0.666	NA	NA
Lymphocyte percentage	0.961 (0.932–0.991)	0.011 *	0.984 (0.941–1.03)	0.492
MLR	7.43 (1.02–54.0)	0.047 *	3.94 (0.324–48.0)	0.282
PLR	45.4 (0.729–2828)	0.070	NA	NA
**Day 3**				
WBC	0.999 (0.885–1.13)	0.985	NA	NA
ALC	1.00 (0.999–1.00)	0.561	NA	NA
Lymphocyte percentage	0.979 (0.953–1.01)	0.115	NA	NA
MLR	18.8 (1.13–315)	0.041 *	-	-
PLR	1.33 (0.075–23.6)	0.848	NA	NA
**Day 4**				
WBC	0.961 (0.882–1.05)	0.369	NA	NA
ALC	1.00 (0.999–1.00)	0.763	NA	NA
Lymphocyte percentage	0.990 (0.960–1.02)	0.507	NA	NA
MLR	2.43 (0.458–12.9)	0.297	NA	NA
PLR	3.26 (0.045–238)	0.590	NA	NA
**Day 5**				
WBC	0.790 (0.675–0.925)	0.003 *	-	-
ALC	0.999 (0.998–1.00)	0.060	NA	NA
Lymphocyte percentage	1.03 (0.988–1.08)	0.161	NA	NA
MLR	3.44 (0.527–22.5)	0.197	NA	NA
PLR	14.0 (0.018–11,112)	0.438	NA	NA

WBC, white blood cell count; ALC, absolute lymphocyte count; MLR, monocyte-to-lymphocyte ratio; PLR, platelet-to-lymphocyte ratio; NA, not applicable. * Denotes statistical significance.
